# Comparison of two ovulation tests to predict timing of the late follicular phase for menstrual cycle research in premenopausal females

**DOI:** 10.14814/phy2.70325

**Published:** 2025-04-22

**Authors:** Lindsay A. Lew, Kyra E. Pyke

**Affiliations:** ^1^ School of Kinesiology and Health Studies, Queen's University Kingston Ontario Canada

**Keywords:** estrogen, late follicular phase, menstrual cycle, ovulation test

## Abstract

Menstrual cycle peak estradiol occurs in the late follicular (LF) phase just prior to the luteinizing hormone (LH) surge and ovulation. Therefore, to examine the impact of menstrual cycle estradiol fluctuations, it is desirable to perform assessments closely prior to ovulation. Standard ovulation tests (SOT) identify the LH surge and confirm that ovulation occurred after LF testing. Advanced ovulation tests (AOT) detect a rise in estrogen before the LH surge. We hypothesized that using the AOT to schedule LF testing between the rise in estradiol and LH surge would decrease the LF visit:ovulation interval vs. the SOT. Twenty‐one naturally menstruating females (22 ± 4 years) participated in an early follicular (EF) and LF visit. The LF visit scheduling employed an AOT (*n* = 10) or SOT (*n* = 11). There was no difference in the LF visit:ovulation interval between tests (AOT = 2.7 ± 2.2 days, SOT = 2.5 ± 1.7 days; *p* = 0.859). Estradiol increased from the EF to LF phase, regardless of the ovulation test used (phase *p* < 0.001, test *p* = 0.528, interaction *p* = 0.099), and Δestradiol was negatively correlated with LF visit:ovulation interval (*r* = −0.454, *p* = 0.050). In this preliminary study, the AOT estrogen signal did not support scheduling the LF visit closer to ovulation or during higher estradiol vs. the SOT. Future studies should explore different methods to identify the menstrual cycle estradiol peak.

## Graphical abstract

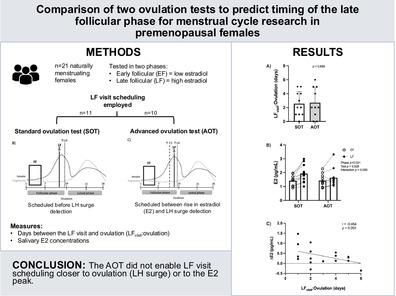

## 1 INTRODUCTION

The early follicular (EF; low estrogen) phase of the menstrual cycle occurs upon the onset of menstruation, and the late follicular (LF; high estrogen) phase extends from the rise in estrogen until its peak just prior to ovulation. These phases are commonly used in experimental models to assess the impact of natural fluctuations in estrogen, as progesterone levels remain low during these phases (Sims & Heather, 2018). Although the menstrual cycle can be a physiologically relevant paradigm for hormonal research, the hormonal fluctuations are also often cited as the rationale to exclude females from physiology research, contributing to historic and current underrepresentation (Lew, Williams, et al., 2022). To ensure an accurate understanding of the physiological impacts of menstrual phase to appropriately inform research decisions, it is important to refine methodology for menstrual phase identification.

The current best practice to assess the timing of the LF phase includes a three‐step method: (1) estimate the day of ovulation from previous cycle length, (2) use an ovulation test to ensure ovulation occurs following the experimental visit, and (3) quantify hormones to confirm the expected rise in estrogen (Williams et al., 2020). Standard ovulation tests (SOT) identify the luteinizing hormone (LH) surge (indicative of ovulation) and only confirm that ovulation occurred after LF testing. However, this is problematic as the retroactive nature of the SOT LH surge indication can lead to long intervals of time between the visit scheduled for LF testing (LF_visit_) and ovulation due to intra‐individual variability in cycle lengths (Liu et al., 2021).

The Clearblue Advanced Ovulation Test (AOT) detects a rise in urinary estrogen (E3G) before the LH surge (Clearblue, 2023) and therefore permits scheduling laboratory testing after a detected rise in estrogen but still before ovulation. This may permit testing closer to the LF phase estrogen peak and thus decrease the LF visit:ovulation interval vs. the SOT. Whether the AOT is beneficial for predicting the estrogen peak prior to ovulation compared to the SOT has not been investigated. Therefore, the purpose of this preliminary study was to investigate whether the AOT permits scheduling LF visits closer to ovulation and thus likely better aligned with the estrogen peak compared to the SOT. We hypothesized that using the AOT, which detects a rise in estrogen prior to the LH surge, would decrease the LF visit:ovulation interval vs. the SOT.

## 2 MATERIALS AND METHODS

### 2.1 Ethical approval

All experimental procedures were approved by the Queen's University Health Sciences and Affiliated Teaching Hospitals Research Ethics Board, which conforms to the standards set by the latest revision of the Declaration of Helsinki (with the exception that this study was not registered in a database). The ethics approval file reference number is 6004461. Before participation, volunteers provided written informed consent on forms approved by the same board.

### 2.2 Participants

Participant data was pooled from two separate study protocols (Lew, Danford, & Pyke, 2022; Liu et al., 2021) that employed SOT and AOT, respectively. The primary outcomes for the original studies were flow‐mediated dilation (Liu et al., 2021) and the hyperemic response to passive leg movement (Lew, Danford, & Pyke, 2022). A total of 21 females were included in this study. Four participants from the original studies by Liu et al. (2021) (*n* = 3) and Lew, Danford, and Pyke (2022) (*n* = 1) were excluded due to a lack of detected ovulation. For both studies, naturally menstruating premenopausal females (age 19–29 years) were recruited from Queen's University and the Kingston community to participate. Volunteers attended a screening visit to consent to participation and determine eligibility. Volunteers completed a medical screening questionnaire, and those who reported use of hormonal contraceptives or irregular menstrual cycles (<10/year) were excluded. To be eligible for participation, volunteers self‐reported no history of cardiovascular and/or metabolic disease, use of prescription medication with possible vasoactive effects, or smoking. Additionally, participants engaged in <5 h/week of moderate‐to‐vigorous physical activity. In the laboratory, height, weight, and blood pressure (BpTRU Medical Devices, Coquitlam, BC, Canada) were measured. Individuals with a BMI >30 kg/m^2^ or who were hypotensive (<90/60 mmHg) or hypertensive (>140/90 mmHg) were excluded.

### 2.3 Experimental design

**FIGURE 1:**
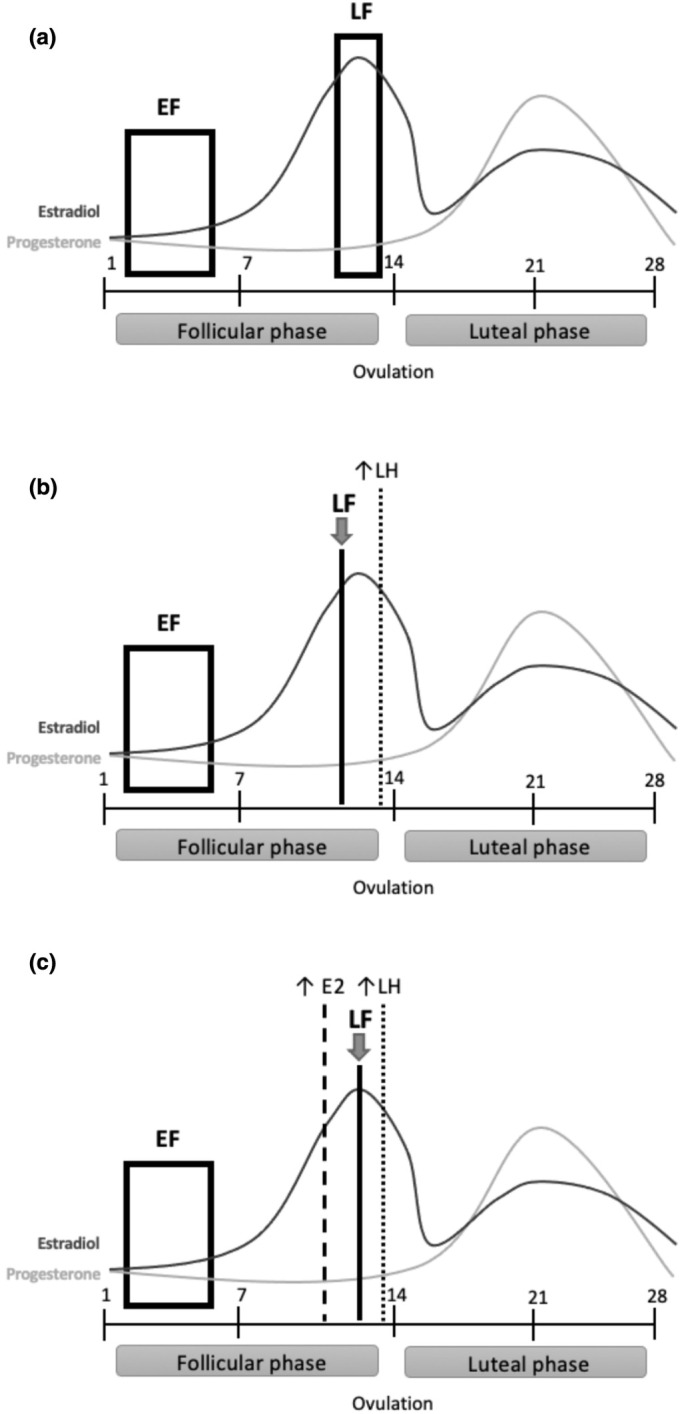
(a) Timing of experimental visits across the menstrual cycle with one visit occurring during the early follicular (EF; low estradiol; Days 2–6) phase and one in the late follicular (LF; high estradiol; just prior to ovulation) phase. (b) Scheduling of LF visit (solid line) before SOT indication of LH surge (dotted line). (c) Scheduling of LF visit (solid line) between AOT indication of rise in estrogen (dashed line) and LH surge (dotted line).

All participants attended two identical experimental visits; one in the EF phase and one in the LF phase (Figure 1a). Prior to experimental visits, participants abstained from food for 12 h, and caffeine, alcohol, and physical activity for 24 h. All visits occurred in the morning, and within participants, visits occurred at the same time of day (±2 h). Each visit included an assessment of salivary estradiol (E2; Salimetrics) along with other procedures reported elsewhere (Lew, Danford, & Pyke, 2022; Liu et al., 2021). Salivary estradiol is moderately to very strongly correlated to blood estradiol (Huang et al., 2023).

### 2.4 Scheduling experimental visits across the menstrual cycle

The EF phase visit occurred 2–6 days following the onset of menstruation. LF visit scheduling employed either an AOT (*n* = 10) using the Clearblue Advanced Digital Ovulation Test (Swiss Precision Diagnostics, Geneva, Switzerland) (Lew, Danford, & Pyke, 2022) or an SOT (*n* = 11) using the Clearblue Ovulation Test (Swiss Precision Diagnostics, Geneva, Switzerland) (Liu et al., 2021). For all participants, the LF visit was initially scheduled at 14–16 days prior to the expected end of the cycle to attempt to time the visit just prior to ovulation. Expected cycle length/end date was based on their immediately previous cycle length. Both ovulation tests required a daily urine sample to detect the concentrations of hormones related to ovulation. Participants were instructed to follow the manufacturer's instructions. The SOT detects when there is a rapid increase in LH, a hormone that triggers ovulation. For participants using the SOT, the LF visit occurred before or on the day of LH surge detection (Figure 1b). The AOT first detects a rise in the urinary metabolite of estrogen and then detects when the LH surge occurs. For participants using the AOT, the LF visit occurred following the rise in estrogen and before or on the day of LH surge detection such that if the rise in estrogen was not detected prior to the LF visit date predicted by cycle length, the visit was delayed until detection of the rise in estrogen (Figure 1c).

### 2.5 Salivary E2 Analysis

Serum 17β‐estradiol levels were assessed by analysis of saliva samples according to the manufacturer's instructions (Salimetrics 17β‐Estradiol Enzyme Immunoassay Kit, Assay #1–3702, State College, PA, USA) as previously described (Lew, Danford, & Pyke, 2022; Liu et al., 2021).

### 2.6 Blood Pressure

Blood pressure (BP) was measured six times and averaged (first measure discarded) with an automated sphygmomanometer (BpTRU BPM‐100; BpTRU Medical Devices). Resting mean arterial pressure (MAP) was calculated with the systolic and diastolic BPTru (BPTru Medical Devices) values using the following equation: MAP = [systolic BP +2 (diastolic BP)]/3 as previously described (Liu et al., 2021).

### 2.7 Statistics

Linear mixed models with factors phase and ovulation test were used to assess group level differences in estradiol. Independent t‐tests were used to assess group level differences in LF_visit_:ovulation interval and other baseline variables between the SOT and AOT. A Pearson's Correlation was used to determine the relationship between LF_visit_:ovulation interval and change in estradiol from the EF to LF phase.

## 3 RESULTS

The analysis included 21 healthy, naturally menstruating premenopausal females (22 ± 4 years), using an AOT (*n* = 10) (Lew, Danford, & Pyke, 2022) or an SOT (*n* = 11) (Liu et al., 2021). This current study is re‐reporting MAP (Liu et al., 2021), LF_visit_:ovulation interval (Liu et al., 2021) and salivary estradiol (Lew, Danford, & Pyke, 2022; Liu et al., 2021) for the subset of participants included from the original studies. There were no differences in age or baseline MAP between groups (Table 1). BMI was higher in the SOT group compared to the AOT group (Table 1).

**TABLE 1:** Participant characteristics.

	SOT (*n* = 11)	AOT (*n* = 10)	*p*‐value
Age (years)	22 ± 4	21 ± 4	0.678
BMI (kg/m^2^)	27.4 ± 4.3	23.1 ± 2.4	0.020*
MAP (mmHg)	79.8 ± 5.9	77.4 ± 5.4	0.353

Abbreviations: AOT, advanced ovulation test; BMI, body mass index; MAP, mean arterial pressure; SOT, standard ovulation test.

* Significant difference between SOT and AOT.

### 3.1 Days between LF visit and ovulation

**FIGURE 2:**
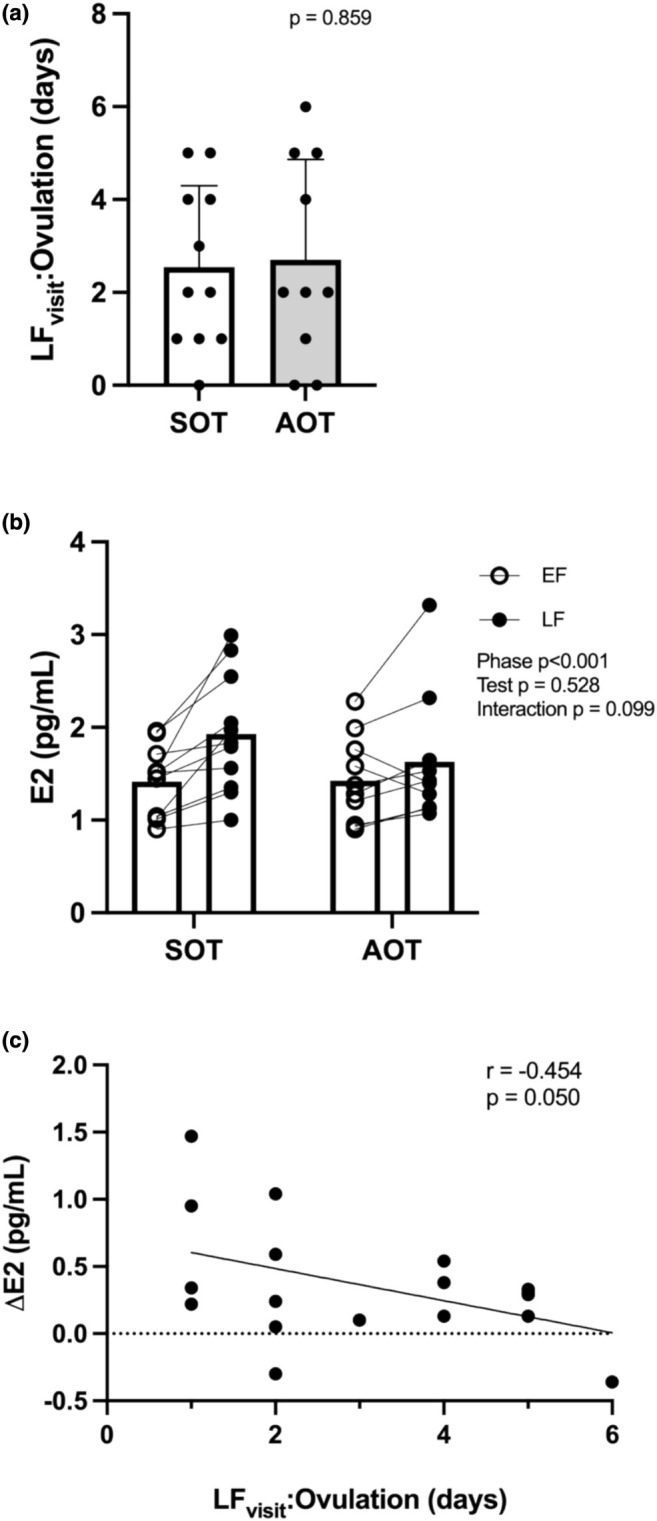
(a) Days between the LF visit and ovulation in participants using the standard (SOT) and advanced ovulation test (AOT). (b) Estradiol in the early (EF) and late follicular (LF) phase when the LF phase visit was scheduled using an SOT or AOT. There was a main effect of phase such that E2 was greater in the LF vs. EF phase. (c) Relationship between LF visit testing and ovulation interval and change in estradiol across the menstrual cycle (LF‐EF).

There was no difference in the LF_visit_:ovulation interval between ovulation tests used (AOT = 2.7 ± 2.2 days, SOT = 2.5 ± 1.7 days; *p* = 0.859; Figure 2a).

### 3.2 Estradiol

Estradiol increased from the EF to LF phase visit, regardless of the ovulation test used (phase *p* < 0.001, test *p* = 0.528, interaction *p* = 0.099; Figure 2b). Change in estradiol from the EF to LF phase was negatively correlated with LF visit:ovulation interval with a zero day interval excluded (*r* = −0.454, *p* = 0.050; Figure 2c) and trending towards significance with all data points included (*r* = −0.380, *p* = 0.080).

## 4 DISCUSSION

This preliminary study was the first to assess whether using an AOT, detecting a rise in estrogen and the LH surge, would allow scheduling of an LF phase visit closer to the estrogen peak and ovulation. Both groups demonstrated an increase in estradiol from the EF to LF phase that was not altered by the type of ovulation test used. Contrary to our hypothesis, we found that there was no difference in the number of days between the LF visit and ovulation (LH surge) when using an AOT vs. an SOT. Importantly, there was a negative relationship between the EF to LF phase Δestradiol and the days between the LF visit and ovulation. This suggests that scheduling experimental visits just prior to ovulation is important to capture peak estrogen levels during the LF phase.

Menstrual cycle research commonly assesses females in the EF and LF phases to understand the impact of endogenous estrogen fluctuations while progesterone remains low. The LF phase encompasses the rise in estrogen that occurs just prior to ovulation and is logistically challenging to capture due to the lack of prospective indication of the desired testing interval (i.e., menstrual bleeding indicating the start of the EF phase). The current standard methodology to assess menstrual phase is the three‐step method, which includes (1) cycle length tracking, (2) confirmation of ovulation, and (3) confirmation of hormone change (Williams et al., 2020). However, despite using this gold standard approach, estimating the timing of the estrogen peak remains challenging due to the high inter‐ and intra‐individual variability in cycle length and thus timing of phases (Grieger & Norman, 2020; Liu et al., 2021). Liu et al. (Liu et al., 2021) assessed naturally menstruating females across two consecutive menstrual cycles and found that cycle length between cycle 1 and 2 was not significantly correlated. Moreover, despite using the well‐established, three‐step methodology for menstrual phase identification, the number of days between the LF laboratory visit and ovulation was not consistent across cycles. Additionally, there was no association between LF phase serum estradiol in cycle 1 and 2, suggesting that there was high inter‐individual variability in estradiol between the LF phase visits.

We found that at the group level estradiol increased from the EF to LF phase. However, there is variability in the change in estradiol at the individual level, with not all participants demonstrating higher estradiol in the LF vs. EF phase visit. This variability is likely due to variable timing of the LF phase visit scheduling compared to the timing of ovulation, with LF visit:ovulation interval ranging from 0 to 6 days. The negative relationship between LF visit:ovulation interval and Δestradiol found in this current study emphasizes the importance of testing just before ovulation to accurately capture the estrogen peak.

The difficulty predicting the LF phase interval at or near the estrogen peak due to inter‐individual variability in cycle length suggests there is a need to reassess the way that menstrual phases are identified in the research setting. Utilizing non‐invasive, immediate, or quick analysis methodologies to track the rise in estrogen is important for the practicality of research design and to allow for enough time for participants to prepare for a laboratory visit (e.g., fasting, and refraining from exercise or alcohol). Although the Clearblue AOT detects a rise in urinary estrogen prior to ovulation, this indication did not improve the ability to predict the timing of the LF phase more proximal to ovulation. The threshold set to indicate a rise in estrogen with the Clearblue AOT may not be high enough to approximate the estrogen peak. Therefore, using the more expensive AOT might not be a worthwhile expense for menstrual cycle researchers.

A limitation of this present study is the small sample size. There was no trend towards a significant change in LF_visit_:ovulation interval days between ovulation tests (p = 0.859) with a small effect size (0.08) suggesting that even a substantially larger sample size would not have revealed a significant difference in the main outcome. Nonetheless, the results of this study must be interpreted with caution due to the preliminary nature of the study design and serve to provide initial methodological insight. Further research on the effectiveness of alternative ovulation prediction strategies is required in larger samples to confirm the findings of this present study.

Future research should explore other non‐invasive options to proactively assess the rise in estrogen to accurately predict the estrogen peak in the LF phase. Continuous urinary or salivary hormone monitoring may provide greater insight into individual variability in hormone fluctuations. Commercial devices (Mira, 2023) or laboratory visits that allow for real‐time daily hormone testing may offer better timing of visits with hormone fluctuation when used in addition to the standard methodologies for assessing menstrual phase. Finding a more reliable way to predict hormone fluctuation within menstrual phase may reduce inter‐study and inter‐individual variability in menstrual cycle research and ensure high‐quality research is being conducted on female participants.

## AUTHOR CONTRIBUTIONS

LAL and KEP were responsible for the conception and design of this study. LAL contributed to data collection and data analysis. LAL and KEP interpreted the data. LAL drafted the paper, with revisions from KEP. All authors reviewed, edited, and approved the final version of the manuscript.

## FUNDING INFORMATION

LAL was supported by an NSERC Postgraduate Scholarship (Doctoral). KEP and LAL are supported by an NSERC Discovery Grant to KEP's Cardiovascular Stress Response Lab.

## CONFLICT OF INTEREST STATEMENT

The authors declare no conflicts of interest.

## Data Availability

Data generated or analyzed during this study are available from the corresponding author upon reasonable request.

## References

[phy270325-bib-0001] Clearblue . (2023). Clearblue advanced digital ovulation test.

[phy270325-bib-0002] Grieger, J. A. , & Norman, R. J. (2020). Menstrual cycle length and patterns in a global cohort of women using a Mobile phone app: Retrospective cohort study. The Journal of Medical Internet Research, 22, e17109. 32442161 10.2196/17109 PMC7381001

[phy270325-bib-0003] Huang, T. , Howse, F. M. , Stachenfeld, N. S. , & Usselman, C. W. (2023). Correlations between salivary‐ and blood‐derived gonadal hormone assessments and implications for inclusion of female participants in research studies. American Journal of Physiology‐Heart and Circulatory Physiology, 324, H33–H46. 36426884 10.1152/ajpheart.00399.2022

[phy270325-bib-0004] Lew, L. A. , Danford, S. , & Pyke, K. E. (2022). Inter‐individual variability in passive leg movement‐induced hyperemia and arterial stiffness across the menstrual cycle: 130. Medicine & Science in Sports & Exercise, 54, 24.

[phy270325-bib-0005] Lew, L. A. , Williams, J. S. , Stone, J. C. , Au, A. K. W. , Pyke, K. E. , & MacDonald, M. J. (2022). Examination of sex‐specific participant inclusion in exercise physiology endothelial function research: A systematic review. Frontiers in Sports and Active Living, 4, 860356. 35399599 10.3389/fspor.2022.860356 PMC8990239

[phy270325-bib-0006] Liu, K. R. , Lew, L. A. , McGarity‐Shipley, E. C. , Byrne, A. C. , Islam, H. , Fenuta, A. M. , & Pyke, K. E. (2021). Individual variation of follicular phase changes in endothelial function across two menstrual cycles. Experimental Physiology, 106, 1389–1400. 33866631 10.1113/EP089482

[phy270325-bib-0007] Mira . (2023). “Mira fertility tracker ‐ accurate fertility tracking and monitoring.” https://www.miracare.com/

[phy270325-bib-0008] Sims, S. T. , & Heather, A. K. (2018). Myths and methodologies: Reducing scientific design ambiguity in studies comparing sexes and/or menstrual cycle phases. Experimental Physiology, 103, 1309–1317. 30051938 10.1113/EP086797

[phy270325-bib-0009] Williams, J. S. , Dunford, E. C. , & MacDonald, M. J. (2020). Impact of the menstrual cycle on peripheral vascular function in premenopausal women: Systematic review and meta‐analysis. American Journal of Physiology‐Heart and Circulatory Physiology, 319, H1327–H1337. 33064553 10.1152/ajpheart.00341.2020

